# Identifying and Characterizing Serious Adverse Drug Reactions Associated With Drug-Drug Interactions in a Spontaneous Reporting Database

**DOI:** 10.3389/fphar.2020.622862

**Published:** 2021-01-18

**Authors:** Lara Magro, Elena Arzenton, Roberto Leone, Marilisa Giustina Stano, Michele Vezzaro, Annette Rudolph, Irene Castagna, Ugo Moretti

**Affiliations:** Section of Pharmacology, Department of Diagnostics and Public Health, University of Verona, Verona, Italy

**Keywords:** drug-drug interactions, spontaneous reporting database, pharmacovigilance, adverse drug reactions, seriousness, polytherapy

## Abstract

**Background:** Drug-drug interactions (DDIs) are an important cause of adverse drug reactions (ADRs). In literature most of studies focus only on potential DDIs, while detailed data on serious ADRs associated with DDIs are limited. Our aim is to identify and characterize serious ADRs caused by DDIs using a spontaneous reporting database.

**Methods:** All serious ADR reports, not related to vaccines and with a “definite”, “probable” or “possible” causality assessment, inserted into the National Pharmacovigilance database from Veneto Region (January 1, 2015 to May 31, 2020) were analyzed. A list of drug pairs was created by selecting the reports containing at least two suspected or concomitant drugs. We verified which drug pairs potentially interacted according to the online version of DRUGDEX^®^ system. For each potential DDI we controlled whether the ADR description in the report corresponded to the interaction effect as described in Micromedex. A detailed characterization of all serious reports containing an occurring DDI was performed.

**Results:** In the study period a total of 31,604 reports of suspected ADRs from the Veneto Region were identified, of which 2,195 serious reports (6.9% of all ADR reports) containing at least two suspected or concomitant drugs were analyzed. We identified 1,208 ADR reports with at least one potential DDI (55.0% of 2,195) and 381 reports (17.4% of 2,195 reports) with an occurring ADR associated with a DDI. The median age of patients and the number of contraindicated or major DDIs were significantly higher in reports with an occurring DDI. Warfarin was the most frequently reported interacting drug and the most common ADRs were gastrointestinal or cerebral hemorrhagic events. The proton pump inhibitors/warfarin, followed by platelet aggregation inhibitors/warfarin were the drug-drug combinations most frequently involved in ADRs caused by DDIs. The highest proportion of fatal reports was observed with platelet aggregation inhibitors/warfarin and antidepressants/warfarin.

**Conclusion:** Our findings showed that about one-third of patients exposed to a potential DDI actually experienced a serious ADR. Furthermore, our study confirms that a spontaneous reporting database could be a valuable resource for identifying and characterizing ADRs caused by DDIs and the drugs leading to serious ADRs and deaths.

## Introduction

The increasing multimorbidity and the rising numbers of guidelines focusing on how to treat specific medical conditions have led to widespread polypharmacy, especially in the elderly. This, together with the ever growing complexity of therapeutic agents available leads to potential drug-drug interactions (DDIs) and consequently to the increased need for investigating the correlated clinical effects, which are still unknown or insufficiently characterized. Adverse drug reactions (ADRs) related to DDIs are an important cause of drug-related problems. Over the past 30 years more than 15,000 articles addressing the drug-drug interactions have been published and to date more than 2,500 pairs of interacting drugs are known. However, in literature most of studies focus only on potential DDIs and depending on the different fields of study and the methodology used, the risk of potential interactions has extremely variable incidence data which range from 6 to 89%, (Solberg et al., 2004; Buurma et al., 2006; Straubhaar et al., 2006; Fitzmaurice et al., 2019). Studies concerning interactions resulting in a clinical issue are fewer and often related to the hospital setting. The incidence of occurring interactions is much lower than that of potential interactions (Magro et al., 2012; Zheng et al., 2018). In general, 68–70% of potential interactions require clinical attention, 1–2% are life-threatening, 17–19% may result in therapeutic benefit and 10–12% have no clinical relevance (Köhler et al., 2000). Up to 14% of ADRs in the hospital setting have been caused by DDIs (Sánchez Muñoz-Torrero et al., 2010), although the clinical relevance of multiple DDIs and their actual clinical importance may be underestimated. A spontaneous reporting database could be an appropriate and valid source to identify, characterize and to quantify the suspected ADRs resulting from DDIs (Van Puijenbroek et al., 2000; Thakrar et al., 2007; Hult et al., 2020). This was the starting point for a previous article in which we described the percentage and characteristics of patients exposed to a potential DDI who experienced a related ADR by analyzing an Italian spontaneous reporting database (Leone et al., 2010), though without focusing on the seriousness of DDI. In the past, fatal drug interactions have been reported in literature (Ferslew et al., 1998; Buajordet et al., 2001; Hung et al., 2005) and some drugs have even been withdrawn from the market because of serious adverse reactions associated with DDIs (Kraft and Waldman, 2001; Wienkers and Heath, 2005). Nevertheless, up to date detailed and targeted data on serious ADRs associated with DDIs are limited. Some authors quantified the importance of drug-drug interactions in the occurrence of ADRs in a pharmacovigilance database, focusing in particular on serious events related to DDIs (Mirošević Skvrce et al., 2011; Montastruc et al., 2012). The aim of the present study is to assess and characterize serious ADRs caused by DDIs using a spontaneous reporting database as data source.

## Materials and Methods

### Data Source

In Italy pharmacovigilance activities are coordinated by the Italian Medicine Agency, which in 2001 developed the national pharmacovigilance database (RNF, *Rete Nazionale di Farmacovigilanza*). RNF allows the collection, management and analysis of all Italian reports of suspected adverse reactions to drugs and vaccines according to the latest European Directive 2010/84/EU. Drugs and reactions are classified according to the Anatomical Therapeutic Chemical Classification (ATC) and to the Medical Dictionary for Regulatory Authorities Activities (MedDRA) respectively (MedDRA, 2020; World Health Organization Collaborating Center for Drug Statistics and Methodology, 2020). An adverse drug reaction is internationally defined as serious when it “results in death, is life-threatening, requires hospitalization or prolongation of existing hospitalization, results in persistent or significant disability or incapacity, or is a congenital anomaly/birth defect” (European Medicine Agengy, 2001). The Naranjo algorithm and the WHO criteria are respectively used to perform the causality assessment of drugs and vaccines (Naranjo et al., 1981; World Health Organization, 2018). The causality assessement refers to the case level according to the Italian national guidelines (Italian Medicines Agency, 2018). Furthermore, the Italian reporting form includes a specific section *Report’s Characteristics* where the reporter can specify if the ADR was caused by medication errors, abuse/misuse, off-label use, overdose, occupational exposure, or drug-drug interaction. This analysis refers to the reports inserted into RNF from Veneto Region which were fully accessible.

### Data Analysis

All serious ADR reports entered, between January 1, 2015 and May 31, 2020, from the Veneto Region not related to vaccines and with a “definite,” “probable” or “possible” causality assessment were analyzed. All reports containing at least two drugs reported as suspected or concomitant were selected, thus creating a list of drug pairs.

The analysis was performed using VigiSegn (Sabaini, 2015), a data warehousing web application for the analysis of the ADR reports collected in RNF. The application has been developed by the Information Technology Team of the Pharmacology Unit in collaboration with the Computer Science Department at the University of Verona for the Veneto Regional Pharmacovigilance and the Italian Medicines Agency. It is based on an open source business intelligence server called Pentaho. The VigiSegn system has an underlying database and OLAP technology (online analytical processing) which can be accessed by means of a high number of queries, reports or dashboards. All the queries have been carefully designed to extract a specific set of data and to perform many types of analysis (Golfarelli and Rizzi, 2009). The ADRs encoded into reports were selected as Preferred Term level.

Potential DDIs were identified using the online version of DRUGDEX^®^ Thomson Micromedex system for drug interaction’s classification and assessment (DRUGDEX Systems, 2020), which is accessible free of charge to online Micromedex^®^ products subscribers. DDIs were classified at four levels of severity according to DRUGDEX^®^ system: 1) contraindicated (the drugs are contraindicated for concurrent use); 2) major (the interaction may be life-threatening and/or require medical intervention to minimize or prevent serious adverse effects); 3) moderate (the interaction may result in an exacerbation of the patient’s condition and/or require an alteration in therapy); and 4) minor (the interaction would have limited clinical effects).

For each potential DDI we verified whether the ADR description in the report corresponded to the interaction effect as described in Micromedex. In case of doubt the assessment was conducted by a panel of experts which included two pharmacologists and two pharmacists, who reached a decision by considering mainly the pharmacological mechanism and the clinical effects of the involved drugs.

A detailed characterization of all serious reports containing an actual/occurring DDI was performed.

## Results

### Case Selection

From January 1, 2015 to May 31, 2020 a total of 31,604 reports of suspected ADRs from the Veneto Region were inserted into RNF, corresponding to 10.9% of the national reports in the same period (Total Number of cases in RNF = 289,001). Veneto population represents 8.1% of the Italian population. In 2019 Veneto ADR reporting rate (RR) was of 1,089 reports per million inhabitants, very close to the national RR (1,078 per million inhabitants).

**FIGURE 1:**
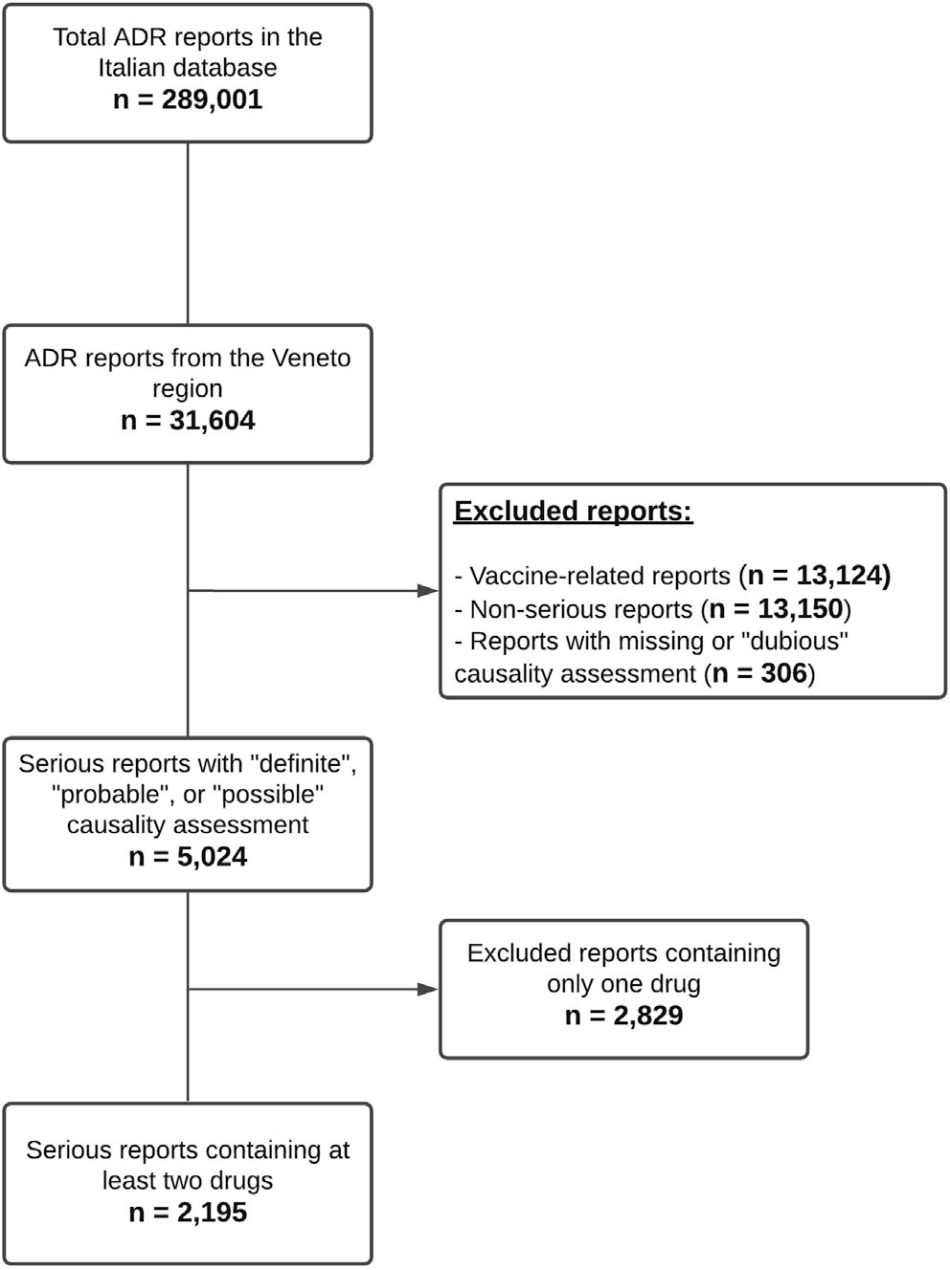
Flowchart of case selection.

As reported in Figure 1 5,024 reports with a causality assessment of “definite,” “probable,” or “possible” were retrieved after excluding vaccine-related (*n* = 13,124) and non-serious reports (*n* = 13,150). Subsequently, 2,829 reports containing only one drug were excluded and a total of 2,195 serious reports containing at least two suspected or concomitant drugs remained for further analysis (6.9% of all ADR reports). In the 2,195 reports (55.6% involving females) a total of 14,473 different drug-drug combinations were identified, of which 1,369 (9.5%) associated with potential DDIs were categorized according to DRUGDEX^®^ as follows: 873 major DDIs (63.8%), 445 moderate DDIs (32.5%), 31 minor DDIs (2.3%) and 20 contraindicated DDIs (1.5%). These potential DDIs were actually reported in 1,208 ADR forms (55.0% of 2,195), in detail: 469 reports contained only one potential DDI, 199 reports contained two potential DDIs and the remaining 540 reports contained more than two potential DDIs.

**FIGURE 2:**
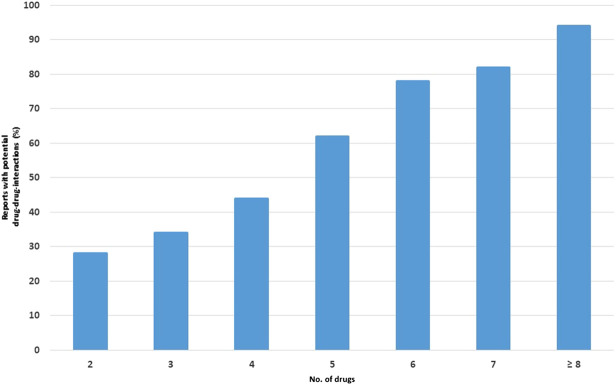
Percentage of reports with potential drug-drug interactions (DDIs) in relation to the number of drugs.

A strong correlation between the number of potential DDIs and the number of concomitantly administered drugs was observed, ranging from 28% in reports containing two drugs to 95% in reports containing eight or more drugs (Figure 2).

### Report’s Characteristics

The analyzed ADR reports were divided into three subgroups: 381 reports (17.4% of 2,195 reports) describing an occurring ADR associated with a DDI (Group A), 827 reports (37.7%) containing a potential DDI (Group B) and 987 reports (45%) containing at least two non-interacting drugs (Group C). Table 1 summarizes the main features of these subgroups.

**TABLE 1:** Main features of reports describing an adverse drug reaction (ADR) associated with an occurred drug-drug interaction (DDI) [Group A], a potential DDI [Group B] or related to patients treated with at least two non-interacting drugs [Group C].

Features of reports	Group A (n = 381)	Group B (n = 827)	Group C (n = 987)
Patient age [yr, mean (95% CI)]	76.1 (74.5–77.8)	65.0 (63.9–66.1)	60.3 (59.0–61.6)
Males [% (95% CI)]	47.0 (42.0–52.0)	43.9 (40.5–47.3)	41.5 (38.5–44.6)
Source of reports [% (95% CI)]			
physicians	90.6 (87.2–93.1)	89.0 (86.7–90.9)	82.7 (80.2–84.9)
pharmacists	4.5 (2.8–7.0)	3.4 (2.3–4.8)	4.9 (3.7–6.4)
other healthcare professionals	3.4 (2.0–5.7)	4.6 (3.3–6.2)	8.6 (7.0–10.5)
citizens	1.5 (0.7–3.4)	3.0 (2.1–4.4)	3.8 (2.8–5.2)
Fatal [% (95% CI)]	4.5 (2.8–7.0)	3.7 (2.6–5.3)	3.2 (2.3–4.5)
No. of drugs [mean (95% CI)]	6.8 (6.4–7.2)	6.7 (6.3–7.2)	3.3 (3.1–3.4)
Contraindicated and major interactions [% (95% CI)]	87.9 (84.3–90.8)	71.4 (68.2–74.3)	Not applicable

The reports of subgroup A represent 1.2% of all Veneto ADR reports and 31.5% of 1,208 reports regarding patients exposed to a potential DDI. Characteristics such as the median age of patients, the number of males, the physicians being the reporter and the fatal cases, were higher in Group A compared to the other groups, but only the patient’s age was significantly different. The percentage of reports with serious criteria such as “hospitalization or prolongation of hospitalization” was higher in Group A if compared to Groups B and C (48.0 *vs* 46.1 *vs* 47.6). The number of concomitantly administered drugs was similar in groups A and B, but was significantly lower in group C. In Group A adult male patients with an occurring ADR caused by DDIs took more drugs than female patients in the same age groups (18–44 years age: 5.2 vs 4.6; 45–64 years age: 8.0 vs 7.9; >64 years age: 7.0 vs 6.7).

Finally, the number of contraindicated and major DDIs was significantly higher in Group A than in Group B.

### Characteristics of Drugs Most Frequently Involved in DDI-Related ADRs

A total of 335 different drug-drug combinations were identified in 381 reports describing an ADR actually caused by a DDI. Table 2 shows the drug-drug combinations reported in at least eight reports as well as the most frequently occurring ADRs and the description of the DDIs as defined by DRUGDEX^®^.

**TABLE 2:** Drug pairs with more than 8 reports of adverse drug reactions (ADRs) (Preferred Term Level) corresponding to the potential interaction effect.

Drug combination (no. of reports)	Most relevant reported ADRs corresponding to the potential interaction effect (no. of reports)	Potential interaction effect (according to DRUGDEX)	Identification of both drugs as suspected by the reporter (no. of reports)	Suspected drugs flagged as interacting in section *Report’s Characteristics* (no. of reports)	
Lansoprazole/warfarin (28)	Increased international normalized ratio [INR] (13), gastrointestinal haemorrhage (10), cerebral haemorrhage (1), conjunctival haemorrhage (1), haematoma (1), haematuria (1)	Elevations in International normalized Ratio serum values and potentiation of anticoagulation effects	1	0	
Aspirin/clopidogrel (18)	Gastrointestinal haemorrhage (11), haematoma (3), cerebral haemorrhage (2), haematuria (1)	Increased risk of bleeding	12	7	
Aspirin/warfarin (18)	Gastrointestinal haemorrhage (8), cerebral haemorrhage (5), haematoma (2)	Increased risk of bleeding	14	7	
Allopurinol/warfarin (16)	Gastrointestinal haemorrhage (6), increased international normalized ratio (4), haematuria (3), cerebral haemorrhage (1), haematoma (1)	Increased INR	0	0	
Omeprazole/warfarin (16)	Increased international normalized ratio (6), gastrointestinal haemorrhage (5), cerebral haemorrhage (1), haematoma (1), mediastinal haemorrhage (1)	Elevations of International normalized Ratio serum values and potentiation of anticoagulant effects	0	0	
Aspirin/furosemide (12)	Acute renale failure (5)	Reduced diuretic effectiveness and possible nephrotoxicity	1	1	
Clopidogrel/warfarin (11)	Gastrointestinal haemorrhage (6), cerebral haemorrhage (2), increased international normalized ratio (3)	Increased risk of bleeding	6	4	
Digoxine/furosemide (10)	Bradycardia (5)	Digoxin toxicity (nausea, vomiting, cardiac arrhythmias)	0	0	
Acetaminophen/warfarin (9)	Gastrointestinal haemorrhage (4), cerebral haemorrhage (1), haemorrhage (1), increased international normalized ratio (1), mediastinal haemorrhage (1)	Increased risk of bleeding	1	1	
Quetiapine/warfarin (9)	Gastrointestinal haemorrhage (4), cerebral haemorrhage (1), haematuria (1), increased international normalized ratio (3)	Increased INR and risk of bleeding	0	0	
Trazodone/warfarin (9)	Gastrointestinal haemorrhage (5), cerebral haemorrhage (1), international normalized ratio increased (3)	Increased risk of bleeding	0	0	
Enoxaparin/warfarin (8)	Gastrointestinal haemorrhage (4), arterial haemorrhage (1), haematuria (1), intra-abdominal haematoma (1)	Increased risk of bleeding	5	2	

Warfarin was the most frequently reported interacting drug and the most common ADRs were gastrointestinal or cerebral hemorrhagic events. In Group A, the drugs actually causing the DDI were recognized and indicated as suspected by reporters in 107 reports out of 381 (28.1%) and the suspected drugs were flagged as interacting in 71 out of 381 reports (18.6%); the most frequently reported drug-drug combinations recognized as suspected were aspirin/clopidogrel and aspirin/warfarin. Table 3 summarizes the most frequently occurring DDIs, grouped by pharmacological classes. The proton pump inhibitors/warfarin, followed by platelet aggregation inhibitors/warfarin were the drug-drug combinations most involved in ADRs caused by DDIs. The highest proportion of fatal reports was observed with platelet aggregation inhibitors/warfarin and antidepressants/warfarin.

**TABLE 3:** Number of ADR reports and fatal reports caused by drug-drug interactions (DDIs).

Drug 1	Drug 2	No. of total reports (%)	Fatal reports *n* (%)
Proton pump inhibitors	Vitamin K antagonists	52	2 (3.8)
Antiplatelet drugs, excl. heparin	Vitamin K antagonists	26	3 (11.5)
Antiplatelet drugs, excl. heparin	Antiplatelet drugs, excl. Heparin	25	1 (4)
Antidepressants	Vitamin K antagonists	18	2 (11.1)
Antidepressants	Antiplatelet drugs, excl. Heparin	17	0
Antigout preparations	Vitamin K antagonists	16	1 (6.3)
NSAIDs^a^	Antiplatelet drugs, excl. Heparin	14	1 (7.4)
Antiplatelet drugs, excl. heparin	Heparin group	12	0
Antiplatelet drugs, excl. heparin	DOACs^b^	12	1 (8.3)
Antiplatelet drugs, excl. heparin	High-ceiling diuretics	12	1 (8.3)

^a^ NSAIDs = Non Steroidal Anti-Inflammatory Drugs.

^b^ DOACs = Direct-Acting Oral Anticoagulants.

The interacting drugs are grouped by pharmacological classes. The table considers DDIs with at least 12 reports.

## Discussion

Polytherapy may determine a cascade effect since it increases the complexity of therapeutic management: the risk of potential DDIs grows proportionally with the number of drugs used and may result in the development of ADRs (Franceschi et al., 2004; Johnell and Klarin, 2007; Siniscalchi et al., 2011). Indeed, our results indicate that there is a strong positive linear relationship between the number of dispensed drugs and the probability of occurrence of a potential DDI, which is in line with previous research (Cadieux, 1989; Köhler et al., 2000; Leone et al., 2010).

Drug-drug interactions are one of the most common causes of ADRs, but their clinical impact in the population has not been sufficiently investigated yet. The aim of this study is to identify and characterize serious ADRs caused by DDIs using a spontaneous reporting database. In our dataset we estimated that 1.2% of all patients experienced a serious ADR associated with a DDI. In literature most of the studies investigating ADR associated with DDIs were conducted in the hospital setting (Davies et al., 2009; Reis and Cassiani, 2011). Detailed data on DDI-associated ADRs for serious reports on suspected ADRs in a spontaneous reporting database are limited. Some authors quantified the occurrence of serious ADRs related to DDIs in a pharmacovigilance database with an estimated range between 56.9 and 74.6% (Tavassoli et al., 2007; Montastruc et al., 2012). These researches varied in sample size and DDI types evaluated from our study. On the other hand, Mirosevic Skvrce and coll. used a similar methodology as ours and found that 2.5% of all patients experienced a serious ADR associated with a DDI (Mirošević Skvrce et al., 2011).

In our study the percentage of patients exposed to a potential DDI who experienced an ADR is consistent with Mirošević Skvrce and coll. results (Mirošević Skvrce et al., 2011). They identified 468 reports containing at least one potential DDI in the Spontaneous Reporting database of the Croatian Agency for Medicinal Products and Medical Devices. Of these, 94 (20.8%) DDIs were related to ADRs, a number which is in line with our results of 31.5%. Tavassoli and coll. reached similar results of 31.4% (Tavassoli et al., 2007).

These findings are consistent with our previous study performed in a spontaneous reporting database analyzing five Italian regions where we had identified 1,159 reports describing an ADR associated with a DDI, corresponding to 2.6% of all reports in the used database and to 21.7% of reports regarding patients exposed to a potential DDI (Leone et al., 2010).

When comparing the characteristics of the patients from the various groups, it is worth noting that in A group, patients were older (76.1 years vs 65.0 years vs 60.3 years), and more exposed to contraindicated and major interactions (87.9% vs 71.4%); furthermore there was a higher number of males (47.0% vs 43.9% vs 41.5%). Additionally, our findings showed that reports related to an ADR associated with a DDI were more frequently fatal (4.5% vs 3.7% vs 3.2%). Among the above-mentioned data, only the age and the number of contraindicated and major DDIs were significantly higher.

According to literature, there is a clear proportional relationship between polypharmacy and age. About 90% (95% CI, 84–93) of elderly patients, mostly aged from 60 to 79 years old take more than five medications and had also the highest incidence rates of consuming more than nine medications [35% (95% CI, 28–41)] (Al Ameri et al., 2014; Midão et al., 2018; Rawle et al., 2018).

Polypharmacy in the elderly population increases the risk of serious adverse drug reactions, occurrence of DDIs and mortality. In addition to this, the physiological changes affecting the pharmacokinetics and pharmacodynamics of many drugs, as well as the poor compliance due to cognitive impairment or depression might place these patients at a greater risk of experiencing ADRs. Furthermore, an ADR may be not promptly recognized, which may lead to additional prescriptions to treat the arising symptoms and consequently increase the risk of further drug-drug interactions (prescribing cascade). Other known risk factors such as the presence and type of previous and/or concomitant pathologies in elderly people need to be considered.

Furthermore, in our study elderly male patients with occurred DDI were more likely to be exposed to polypharmacy than female patients. A similar finding was found in literature (Al Ameri et al., 2014; Santalucia et al., 2015).

With regard to the differences within the three groups between contraindicated, major interactions and fatal reports, it is important to underline that health care professionals have been dealing with problems related to serious ADR caused by DDIs for many years. In the early 1990s some case reports of fatal *torsade de pointes* induced by the accumulation of terfenadine, whose metabolism was inhibited by coadministration of ketoconazole and erythromycin were published (Monahan et al., 1990; Mathews et al., 1991; Woosley et al., 1993). This problem contributed to the drug’s withdrawal in 1998 and these case reports drew attention on the clinically relevant implications of serious occuring DDIs. The clinical relevance of drug interactions is demonstrated also by being a predictor of ADR-related hospital admission in the elderly people (Franceschi et al., 2008; Kovačević et al., 2019). In the current study 4.5% of patients experienced fatal DDIs, a percentage consistent with the 4.2% found in our previous study (Leone et al., 2010). In literature, most of the evidence about fatal ADRs caused by DDIs comes from case reports or case series (Moling et al., 2009; Magee et al., 2010). These data suggest that DDIs may play a major role in inducing severe ADRs, thus the necessity to carefully evaluate them, particularly in elderly subjects who need polypharmacy.

Our analysis showed many cases of bleeding deriving from the use of warfarin associated with anti-platelet agents, antidepressants, allopurinol and proton-pump inhibitors, with consequent important percentage of fatal reports (11.5, 11.1, 6.3, and 3.8% respectively). These interactions are well known and documented in literature. Bleeding is a common complication of warfarin therapy and its hepatic metabolism and synergism are the most common underlying mechanisms for the occurrence of drug-drug interactions. The co-administration of these types of drugs requires great caution since it may result in serious ADRs, as it has been previously reported by other authors (Blix et al., 2004; Mirosevic Skvrce et al., 2011; Spina et al., 2020). In patients starting therapy with warfarin, physicians should consider using an alternative medication with a more limited potential for interactions and provide recommendations for the timing of INR monitoring.

In our study the risk of bleeding was caused also by the combination of two different platelet aggregation inhibitors such as aspirin and clopidogrel (Payne et al., 2002; Suzuki et al., 2015).

The interaction between digoxin and furosemide (see Table 2) is one of the most frequently reported DDIs in our study. This finding is in line with two previous analyses conducted by our group in two different settings: a pharmacovigilance database and a general practitioner database, which showed that notwithstanding well-known drug interactions caused by digoxin’s narrow therapeutic index, physicians sometimes use intentionally digoxin in combination with furosemide (Magro et al., 2008; Leone et al., 2010).

The reporters recognized both interacting drugs as suspected only in 28.1% of the reports. Furthermore, also the single percentage of recognition and the inclusion of a flag related to some DDIs such as omeprazole/warfarin was null. On the other hand, reporters have shown more confidence with some DDIs such as warfarin/aspirin (78%) and aspirin/clopidogrel (67%). These findings are in accordance with previous results (Tegeder et al., 1999; Leone et al., 2010; Abdo et al., 2020). Increased awareness of the major DDIs among reporters may enhance their recognition rate and help to prevent and avoid them. A drug-drug interaction is often predictable and therefore avoidable or well manageable (Johnell and Klarin, 2007; Ismail et al., 2011). Many of the serious occurring ADRs due to DDIs we identified could have probably been avoided by monitoring the patient more closely or by using alternative medications. The use of drugs should be reviewed regularly and, when possible, unnecessary agents should be withdrawn. Since it is not realistic to expect health care professionals to recollect all possible interactions related to every medication their patients take, the use of several resources may assist them in recognizing and consequently avoiding DDIs. In a prospective controlled intervention cohort study the effect of electronic drug information on the incidence of potential and actual DDIs in intensive care patients was investigated. The number of patients with at least one potential DDI decreased by 18% and the number of actual DDIs decreased by 43% (Bertsche et al., 2010).

Our study confirmed the spontaneous reporting database as a valuable resource for identifying and characterizing ADRs caused by DDIs and the drugs leading to serious ADRs and deaths, thus emphasizing the areas where health care education should focus.

Our study shares important limitations typical of the spontaneous reporting system, first of all the under-reporting of ADRs. A systematic review estimated a median under-reporting rate of 94% across 37 studies (interquartile range 82–98%) (Hazell and Shakir, 2006). Secondly, the presence in our database of reports with missing information on concomitant drugs and co-morbidity is another limit. Though providing essential information, a spontaneous report database has specific characteristics which are certainly not comparable with personal records. An additional limit is the lack of denominator data such as the user population. Compared to other methodologies, the use of a single source of drug interaction-checking method (Montastruc et al., 2012; Patel and Beckett, 2016), may have hindered the drug-drug interaction identification. On the other hand, since a standardization of both the identification and the severity rating systems of DDIs is lacking, the use of DRUGDEX^®^, a fully referenced comprehensive drug resource with unbiased content on interactions improved the consistency in medication safety, health and disease management.

## Conclusion

Most of the studies in literature have focused on potential DDIs. However, to quantify the clinical impact of drug interactions on public health, only adverse drug reactions associated with DDIs should be considered. The percentage of patients exposed to a potential DDI who experienced a serious ADR of 31.5% confirms that DDIs could be a clinically relevant problem. Our findings show defined and characterized data on the serious and most important consequences of occurring DDIs, the majority of which involve a small number of drugs as warfarin and aspirin. In some situations, a closer monitoring of the patient could prevent serious consequences, while information technology could reduce physicians’ workload and help improving clinical prescription behaviour. Further research should quantify such serious harms more systematically, because understanding when harms outweigh benefits is essential for reaching rational treatment decisions. Clinical guidelines and management strategies regarding the consequences of occurring DDIs should be developed. Furthermore, more educational training for prescribers regarding the knowledge and recognition of serious adverse drug reactions associated with drug-drug interactions should be improved.

## Data Availability Statement

The datasets presented in this article are not readily available because datasets are only available for the Italian Medicine Agency or Pharmacovigilance Regional Centres. Requests to access the datasets should be directed to **lara.magro@univr.it**

## Ethics Statement

Safety data deriving from the Italian spontaneous reporting system are anonymous and in compliance with the ethical standard. Therefore, no further ethical measures were required.

## Author Contributions

RL and UM conceived the study. LM and EA contributed to the study design. Data analysis was curated by LM, EA, MS, MV, AR, and IC. The first draft of the manuscript was written by LM, EA, and AR. RL and UM critically revised the manuscript. All authors read and approved the final manuscript.

## Conflict of Interest

The authors declare that the research was conducted in the absence of any commercial or financial relationships that could be construed as a potential conflict of interest.
